# The “1958 He^4^ Scale of Temperatures”^1^: Part 2. Tables for the 1958 Temperature Scale

**DOI:** 10.6028/jres.064A.002

**Published:** 1960-02-01

**Authors:** H. van Dijk, M. Durieux, J. R. Clement, J. K. Logan

## 1. Introduction

The tables which follow are these:

Table I. *Vapor pressure of* He^4^ (*1958 Scale) in microns (10*^−3^ *mm) mercury at 0*° *C and standard gravity (980.665 cm/sec^2^).* This table is an expanded version, with pressure values at millidegree intervals, of the table which defines the vapor pressure on the 1958 scale at 10-millidegree intervals.
Table II. *1958* He^4^ *vapor pressure-temperature scale*, T *in °K as a junction of* P *in millimeters mercury at 0° C and standard gravity.* This table is an inversion of table I for appropriate ranges of pressures and pressure intervals. Since this table contains differences between successive entries, it furnishes a convenient means for converting a measured vapor pressure to a temperature within 0.1 millidegree.
Table III. *1958* He^4^ *vapor pressure-temperature scale*, T *in °K as a function of* P *in centimeters mercury at 0° C and standard gravity.* This table is an extension of table II for pressures greater than 80 cm mercury. It is numbered independently because the pressure unit is centimeters mercury rather than millimeters mercury.
Table IV. *Temperature derivative in millimeters* Hg/°*K for the 1958* He^4^ *Scale.* This table gives values of the first derivative, *dP/dT*, to four significant digits. These values are smoother and more precise than values obtained directly from table I by simple difference calculation, and they represent true derivatives of the scale defined by table I.
Table V. *Auxiliary table for making hydrostatic head correction.* This table gives, as a function of pressure, values of the ratio between the density of liquid He I and the density of mercury at 0° C.
Table VI. *Deviations of earlier scales from the 1958 scale.* The definitions of earlier scales used for obtaining the values in this table are explicitly given in explanatory notes which accompany it.
Table VII. *Auxiliary table for making corrections for the density of mercury at temperatures other than 0° C.* This table gives values of the ratio between the density of mercury at temperatures between 10° and 39° C and the density at 0° C. Following this table is an equation useful for making another correction which in precise work must be applied to the observed height of a mercury column. This equation gives an empirical relation between the acceleration due to gravity and the local latitude and altitude. If the local value of this acceleration is unknown, the equation will yield a value sufficiently accurate for the purpose of manometry.

## 2. Constants Used in the Computation of the Scale

Certain constants are necessary for computing a vapor pressure-temperature scale. The values of these constants adopted for the computation of the 1958 scale are tabulated below. The significance of the *L*_0_ value may be found in Part 1, Introduction by F. G. Brickwedde. These constants are: *i*_0_ = 12.2440 cgs units; *L*_0_=59.62 j/mole; *R*=8.31662 j/mole·deg; density of mercury at 0° C= 13.5951 g/cm^3^; standard gravity=980.665 cm/sec^2^; pressure at the λ-point = 37.80 mm mercury at 0° C and standard gravity.

## 3. Fixed Points on the Scale

The boiling point is at 4.2150° K for *P*= 760.00 mm mercury at 0° C and standard gravity (or 1013250 dynes/cm^2^). The λ-point is at 2.172(1° K for *P* as noted above. The critical point, if the critical pressure is taken to be 1718 mm mercury (Kamerlingh Onnes, Leiden Comm. 124b) at 0° C and standard gravity, is at 5.1994° K.

## 4. Comments on Determining Temperature by Measuring Vapor Pressure

Two techniques are commonly used for determining a temperature by measuring the vapor pressure of liquid He^4^. In one, the pressure at some point above a bath of liquid helium is measured. In this case, standard practice has been to add to the measured pressure, when above the λ-point pressure, an amount equal to the pressure exerted by the column of helium between the point where the pressure is measured and the point in the bath occupied by the object whose temperature is desired. Below the λ-point, no correction of the observed pressure is ordinarily made, although the pressure drop in the gas due to pumping may become significant at low pressures. In the other technique, the pressure over a small amount of helium condensed in a “vapor pressure bulb” is measured. Since this “bulb” is normally placed close to the point in the bath occupied by the object whose temperature is desired, correction of the observed pressure is usually considered unnecessary above, as well as below, the λ-point. Numerous arrangements have been used for the pressure-transmitting line from the “bulb” to the manometer, but no standard practice seems to prevail. In any such apparatus, thermomolecular pressure differences between the cold “bulb” and the warm manometer arise at sufficiently low pressures.

It is generally known that various adaptations of these two techniques yield slightly different pressures and therefore slightly different temperatures, especially above the λ-point. Although these temperatures usually differ by no more than 0.01° K, special attention to technique seems required when precision exceeding 0.01° K is desired. Two conditions necessary to any satisfactory technique for determining the temperature of an object by measuring the vapor pressure of a liquid seem obvious. First, there must be thermal equilibrium between the object and the liquid. Second, the pressure at which the liquid is in equilibrium with its saturated vapor must somehow be determined. In the case of He^4^ there is one criterion which, if satisfied, probably assures that these conditions are met. Differences between the thermal properties of He^4^ above and below the λ-point are so large that, if the calibration of a secondary thermometer yields a continuous curve through the λ-point, the technique by which the calibration was obtained is probably satisfactory.

## Equation for Computing Local Acceleration Due to Gravity^1^, ^2^

Most frequently the vapor pressure is measured as a distance between two mercury levels. After corrections for capillarity and for the temperature of the mercury and the scale have been applied, the height, *h*, has to be reduced to standard gravity. The reduced height, *h_0_*, can be computed from *h*_0_=980.665. If the local value of *g* is unknown, it may be computed with sufficient accuracy for correcting the height of a mercury column from

g=980.632−2.586cos2ϕ+0.003cos4ϕ−0.0003086H

where *ϕ* is the local latitude and *H* the local altitude in meters. The unit of *g* is cm/sec^2^.

## Figures and Tables

**Table I:** Vapor pressure of *He*^4^ (1958 scale) in microns (10^−3^ mm) mercury at 0° C and standard gravity (980.665 cm/sec^2^)

*T*°K	0.000	0.001	0.002	0.003	0.004	0.005	0.006	0.007	0.008	0.009

0.50	0.016342	0.016901	0.017476	0.018069	0.018680	0.019309	0.019956	0.020623	0.021310	0.022017
.51	.022745	.023494	.024265	.025058	.025875	.026714	.027578	.028467	.029381	.030321
.52	.031287	.032280	.033301	.034351	.035431	.036541	.037681	.038852	.040055	.041291
.53	.042561	.043865	.045205	.046581	.047993	.049443	.050932	.052461	.054030	.055640
.54	.057292	.058987	.060727	.062512	.064343	.066221	.068147	.070123	.072149	.074226
.55	.076356	.078540	.080779	.083074	.085426	.087836	.090306	.092837	.095431	.098088
.56	.10081	.10360	.10646	.10938	.11237	.11544	.11858	.12179	.12508	.12845
.57	.13190	.13543	.13904	.14274	.14652	.15039	.15435	.15840	.16254	.16678
.58	.17112	.17555	.18008	.18471	.18945	.19430	.19926	.20433	.20951	.21480
.59	.22021	.22574	.23139	.23716	.24306	.24908	.25524	.26153	.26795	.27451
0.60	0.28121	0.28805	0.29504	0.30218	0.30947	0.31691	0.32450	0.33225	0.34017	0.34825
.61	.35649	.36490	.37349	.38225	.39120	.40032	.40963	.41912	.42881	.43869
.62	.44877	.45905	.46953	.48023	.49113	.50225	.51358	.52514	.53692	.54894
.63	.56118	.57366	.58638	.59935	.61256	.62603	.63975	.65374	.66799	.68250
.64	.69729	.71236	.72771	.74334	.75926	.77548	.79200	.80882	.82595	.84340
.65	.86116	.87925	.89766	.91641	.93550	.95493	.97471	.99484	1.0153	1.0362
.66	1.0574	1.0790	1.1010	1.1233	1.1461	1.1692	1.1928	1.2167	1.2411	1.2659
.67	1.2911	1.3168	1.3429	1.3694	1.3964	1.4238	1.4518	1 4802	1.5090	1.5384
.68	1.5682	1.5985	1.6293	1.6607	1.6925	1.7249	1.7578	1.7913	1.8253	1.8598
.69	1.8949	1.9306	1.9669	2.0037	2.0412	2.0792	2.1179	2 1571	2.1970	2.2375
.70	2.2787	2.3205	2.3629	2.4061	2.4499	2.4944	2.5395	2.5853	2 6319	2.6792
.71	2.7272	2.7760	2.8255	2.8757	2.9267	2.9785	3.0311	3.0845	3.1386	3.1936
.72	3.2494	3.3060	3.3635	3.4218	3.4810	3.5411	3.6021	3.6639	3.7266	3.7903
.73	3.8549	3.9204	3.9869	4.0544	4.1228	4.1922	4.2626	4.3340	4.4064	4.4798
.74	4.5543	4.6298	4.7064	4.7841	4.8629	4.9428	5.0238	5.1059	5.1891	5 2735
.75	5.3591	5.4459	5.5338	5.6230	5.7134	5.8050	5.8978	5.9919	6.0873	6.1840
.76	6.2820	6.3813	6.4819	6.5839	6.6872	6.7919	6.8980	7.0055	7.1144	7.2247
.77	7.3365	7.4498	7.5645	7.6807	7.7985	7.9178	8.0386	8.1610	8.2849	8.4104
.78	8.5376	8.6664	8.7968	8 9289	9.0627	9.1981	9.3352	9.4741	9.6147	9.7571
.79	9.9013	10.047	10.195	10.345	10.496	10.650	10.805	10.962	11.121	11.282
.80	11.445	11.610	11.777	11.946	12.117	12.290	12.465	12.642	12.822	13.003
.81	13.187	13.373	13.561	13.751	13.944	14.138	14.335	14.535	14.737	14.941
.82	15.147	15.356	15.567	15.781	15.997	16.216	16 437	16.661	16.887	17.116
.83	17.348	17.582	17.819	18.058	18.300	18.545	18.793	19.043	19.296	19.552
.84	19.811	20.073	20.337	20.605	20.875	21.149	21.425	21.704	21.987	22.272
.85	22.561	22.853	23.148	23.446	23.747	24.052	24.360	24.671	24 985	25.303
.86	25.624	25.948	26.276	26.608	26.943	27.281	27.623	27.969	28.318	28.671
.87	29.027	29.387	29.751	30.119	30.490	30.865	31.245	31.628	32.014	32.405
.88	32.800	33.199	33.602	34.009	34.420	34.835	35.254	35.678	36.106	36.538
.89	36.974	37.415	37.860	38.309	38.763	39.221	39.684	40.151	40.623	41.100
.90	41.581	42.067	42.557	43.053	43.553	44.058	44.568	45.082	45.602	46.126
.91	46.656	47.191	47.730	48.275	48.825	49.380	49.940	50.505	51.076	51.652
.92	52.234	52.821	53.414	54.012	54.615	55.224	55.839	56.459	57.085	57.717
.93	58.355	58.999	59.648	60.303	60.964	61.632	62.305	62.984	63.670	64.361
.94	65.059	65.763	66.473	67.190	67.913	68.642	69.378	70.120	70.869	71.624
.95	72.386	73.155	73.930	74.713	75.502	76.298	77.101	77.910	78.727	79.551
.96	80.382	81.220	82.066	82.918	83.778	84 645	85.520	86 462	87.291	88.188
.97	89.093	90.005	90.925	91.853	92.789	93.732	94.683	95.642	96.609	97.584
.98	98.567	99.558	100.557	101.565	102.581	103.605	104.658	105.679	106.728	107.786
.99	108.853	109.928	111.012	112.104	113.205	114.315	115.434	116.562	117.699	118.845
1.00	120.000	121.165	122.339	123.523	124.716	125.918	127.129	128.350	129.580	130.820
1.01	132.070	133.330	134.600	135.880	137.169	138.468	139.778	141.097	142.427	143.766
1.02	145.116	146.477	147.848	149.230	150.622	152.025	153.438	154.862	156.297	157.742
1.03	159.198	160.666	162.145	163.634	165.135	166.647	168.170	169.704	171.250	172.806
1.04	174.375	175.956	177.548	179.152	189.768	182.395	184.635	185.086	187.349	189.024
1.05	190.711	192.412	194.124	195.849	197.587	199.336	201.698	202.874	204.661	206.461
1.06	208.274	210.101	211.941	213.794	215.660	217.538	219.431	221.335	223.255	225.187
1.07	227.132	229.092	231.065	233.052	235.053	231.068	239.096	241.138	243.195	245.265
1.08	247.350	249.450	251.565	253.694	255.838	257.995	260.168	262.355	264.557	266.774
1.09	269.006	271.254	273.516	275.794	278.087	280.396	282.719	285.058	287.413	289.783
1.10	292.169	294.572	296.991	299.426	301.877	304.344	306.828	309.327	311.843	314.375
1.11	316.923	319.489	322.072	324.671	327.287	329.920	332.570	335.237	337.921	340.622
1.12	343.341	346.079	348 834	351.606	354.397	357.205	360.030	362.874	365.735	368.614
1.13	371.512	374.429	377.364	380.318	383.290	386.280	389.290	392.317	395.364	398.430
1.14	401.514	404.619	407.744	410.887	414.050	417.232	420.434	423.655	426.896	430.156
1.15	433.437	436.739	440.060	443.402	446.764	450.146	453.549	456.972	460.416	463.’880
1.16	467.365	470.873	474.402	477.951	481.522	485.114	488.728	492.363	496.019	499.697
1.17	503.396	507.118	510.863	514.630	518.418	522.229	526.062	529.917	533.794	537.694
1.18	541.617	545.564	549.535	553.528	557.544	561.583	565.645	569.731	573.840	577.972
1.19	582.129	586.310	590.515	594.744	598.998	603.275	607.576	611.901	616.251	620.626
1.20	625.025	629.450	633.901	638.377	642.877	647.403	651.954	656.530	661.131	665.758
1.21	670.411	675.091	679.797	684.529	689.287	694.071	698.881	703.717	708.580	713.470
1.22	718.386	723.331	728.303	733.301	738.327	743.380	748.461	753.569	758.704	763.866
1.23	769.057	774.277	779.525	784.801	790.105	795.437	800.798	806.187	811.605	817.052
1.24	822.527	828.033	833.569	839.135	844.730	850.353	856.006	861.689	867.402	873.144
1.25	878.916	884.720	890.555	896.420	902.315	908.241	914.197	920.184	926.202	932.250
1.26	938.330	944.442	950.585	956.759	962.965	969.203	975.473	981.774	988.107	994.472
1.27	1090.87	1007.30	1013.77	1020.27	1026.80	1033.36	1039.95	1046.58	1053.24	1059.94
1.28	1066.67	1073.44	1080.24	1087.07	1093.93	1100.83	1107.77	1114.74	1121.74	1128.78
1.29	1135.85	1142.96	1150.10	1157.28	1164.49	1171.74	1179.02	1186.34	1193.69	1201.08
1.30	1208.51	1215.98	1223.48	1231.02	1238.59	1246.20	1253.85	1261.54	1269.26	1277.02
1.31	1284.81	1292.64	1300.51	1308.42	1316 37	1324 35	1332 37	1340.43	1348.53	1356.66
1.32	1364.83	1373.04	1381.30	1389.59	1397.92	1406 29	1414.70	1423.15	1431.64	1440.16
1.33	1448.73	1457.34	1465.98	1474.67	1483.40	1492.17	1500.97	1509.82	1518.71	1527.64
1.34	1536.61	1545.62	1554.68	1563.78	1572.91	1582.09	1591.31	1600.57	1609.88	1619.23
1.35	1628.62	1638.06	1647.54	1657.06	1666.62	1676.23	1685.88	1695.57	1705.31	1715.09
1.36	1724.91	1734.78	1744.69	1754.65	1764.65	1774.69	1784.78	1794 91	1805.09	1815.31
1.37	1825.58	1835.90	1846.26	1856.66	1867.11	1877.61	1888.16	1898.75	1909.38	1920.06
1.38	1930.79	1941.57	1952.39	1963.26	1974.18	1985.14	1996.15	2007.21	2018.32	2029.47
1.39	2040.67	2051.92	2063.22	2074.57	2085.96	2097.40	2108.89	2120.43	2132.02	2143.66
1.40	2155.35	2167.09	2178.88	2190.72	2202.61	2214.54	2226.53	2238.57	2250.66	2262.80
1.41	2274.99	2287.23	2299.52	2311.87	2324.27	2336.72	2349.22	2361.77	2374.37	2387.02
1.42	2399.73	2412.49	2425.30	2438.17	2451.09	2464.06	2477.09	2490.17	2503.30	2516.48
1.43	2529.72	2543.01	2556.36	2569.76	2583.21	2596.72	2610.29	2623.91	2637.58	2651.31
1.44	2665.09	2678.93	2692.82	2706.77	2720.78	2734.84	2748.96	2763.13	2777.36	2791.65
1.45	2805.99	2820.39	2834.85	2849.37	2863.95	2878.58	2893.27	2918.02	2922.82	2937.68
1.46	2952.60	2967.58	2982.62	2997.72	3012.88	3028.09	3043.36	3058.69	3074.08	3089.53
1.47	3105.04	3120.61	3136.24	3151.94	3167.69	3183.50	3199.37	3215.30	3231.30	3247.36
1.48	3263.48	3279.66	3295.90	3312 20	3328.57	3345.00	3361.49	3378.14	3394.65	3411.33
1.49	3428.07	3444.87	3461.74	3478.67	3495.66	3512.72	3529.84	3547.03	3564.28	3581.59
1.50	3598.97	3616.41	3633.92	3651.49	3669.13	3686.83	3704.60	3722.43	3740.33	3758.29
1.51	3776.32	3794.42	3812.59	3830.82	3849.12	3867.49	3885.92	3904.42	3922.99	3941.62
1.52	3960.32	3979.09	3997.93	4016.83	4035.80	4054.84	4073.95	4093.12	4112.37	4131.69
1.53	4151.07	4170.52	4190.05	4209.65	4229.32	4249.06	4268.87	4288.74	4308.68	4328.70
1.54	4348.79	4368.95	4389.17	4409.47	4429.84	4450.28	4470.80	4491.39	4512.05	4532.78
1.55	4553.58	4574.46	4595.41	4616.44	4637.54	4658.71	4679.96	4701.28	4722.67	4744.14
1.56	4765.68	4787.29	4808.98	4830.74	4852.58	4874.49	4896.48	4918.54	4940.68	4962.89
1.57	4985.18	5007.54	5029.98	5052.50	5075.09	5097.76	5120.51	5143.33	5166.23	5189.21
1.58	5212.26	5235.39	5258.60	5281.89	5305.26	5328.71	5352.23	5375.83	5399.51	5423.27
1.59	5447.11	5471.03	5495.02	5519.10	5543.26	5567.49	5591.81	5616.21	5640.68	5665.24
1.60	5689.88	5714.60	5739.40	5764.28	5789.25	5814.30	5839.42	5864.63	5889.92	5915.30
1.61	5940.76	5966.30	5991.92	6017.62	6043.41	6069.28	6095.24	6121.28	6147.40	6173.61
1.62	6199.90	6226.27	6252.72	6279.26	6305.88	6332.59	6359.39	6386.27	6413.23	6440.28
1.63	6467.42	6494.64	6521.95	6549.35	6576.84	6604.41	6632.07	6659.81	6687.64	6715.56
1.64	6743.57	6771.66	6799.84	6828.11	6856.47	6884.91	6913.44	6942.07	6970.78	6999.58
1.65	7028.47	7057.45	7086.52	7115.67	7144.92	7174.26	7203.69	7233.21	7262.82	7292.52
1.66	7322.31	7352.19	7382.15	7412.21	7442.36	7472.60	7502.94	7533.37	7563.89	7594.50
1.67	7625.21	7656.01	7686.90	7717.88	7748.96	7780.13	7811.40	7842.76	7874.21	7905.76
1.68	7937.40	7969.13	8000.96	8032.88	8064.90	8097.01	8129.22	8161.53	8193.93	8226.43
1.69	8259.02	8291.70	8324.48	8357.36	8390.33	8423.40	8456.57	8489.84	8523.20	8556.66
1.70	8590.22	8623.87	8657.62	8691.47	8725.42	8759.47	8793.62	8827.86	8862.20	8896.64
1.71	8931.18	8965.82	9000.56	9035.40	9070.33	9105.36	9140.50	9175.74	9211.08	9246.52
1.72	9282.06	9317.70	9353.44	9389.28	9425.22	9461.26	9497.41	9533.66	9570.01	9606.46
1.73	9643.02	9679.68	9716.45	9753.32	9790.29	9827.36	9864.54	9901.82	9939.21	9976.70
1.74	10014.3	10052.0	10089.8	10127.7	10165.7	10703.8	10242.0	10280.3	10318.7	10357.3
1.75	10395.9	10434.6	10473.4	10512.4	10551.5	10590.7	10630.0	10669.4	10708.9	10748.5
1.76	10788.2	10828.0	10867.9	10907.9	10948.0	10988.3	11028.7	11069.2	11109.7	11150.4
1.77	11191.2	11232.1	11273.1	11314.2	11355.4	11396.7	11438.1	11479.7	11521.4	11563.2
1.78	11605.1	11647.1	11689.2	11731.4	11773.7	11816.1	11858.7	11901.4	11944.2	11987.1
1.79	12030.1	12073.2	12116.4	12159.7	12203.1	12246.6	12290.3	12334.1	12378.0	12422.0
1.80	12466.1	12510.3	12554.7	12599.2	12643.8	12688.5	12733.3	12778.2	12823.2	12868.4
1.81	12913.7	12959.1	13004.6	13050.2	13095.9	13141.8	13187.8	13233.9	13280.1	13326.4
1.82	13372.8	13419.3	13466.0	13512.8	13559.7	13606.7	13653.8	13701.1	13748.5	13796.0
1.83	13843.6	13891.3	13939.1	13987.1	14035.2	14083.4	14131.7	14180.1	14228.6	14277.3
1.84	14326.1	14375.0	14424.0	14473.2	14522.5	14571.9	14621.4	14671.0	14720.8	14770.7
1.85	14820.7	14870.8	14921.0	14971.4	15021.9	15072.5	15123.2	15174.0	15225.0	15276.1
1.86	15327.3	15378.6	15430.1	15481.7	15533.4	15585.2	15637.2	15689.3	15741.5	15793.8
1.87	15846.3	15898.9	15951.6	16004.4	16057.3	16110.4	16163.6	16216.9	16270.4	16324.0
1.88	16377.7	16431.5	16485.5	16539.6	16593.8	16648.1	16702.6	16757.2	16811.9	16866.7
1.89	16921.7	16976.8	17032.0	17087.3	17142.8	17198.4	17254.1	17309.9	17365.9	17422.0
1.90	17478.2	17534.6	17591.1	17647.7	17704.5	17761.4	17818.4	17875.5	17932.8	17990.2
1.91	18047.7	18105.4	18163.2	18221.1	18279.1	18337.3	18395.6	18454.0	18512.6	18571.3
1.92	18630.1	18689.0	18748.1	18807.3	18866.7	18926.2	18985.8	19045.5	19105.4	19165.4
1.93	19225.5	19285.8	19346.2	19406.7	19467.4	19528.2	19589.1	19650.1	19711.3	19772.6
1.94	19834.1	19895.7	19957.4	20019.2	20081.2	20143.3	20205.6	20268.0	20330.5	20393.1
1.95	20455.9	20518.8	20581.9	20645.1	20708.4	20771.8	20835.4	20899.1	20963.0	21027.0
1.96	21091.1	21155.4	21219.8	21284.3	21348.9	21413.7	21478.6	21543.7	21608.9	21674.2
1.97	21739.7	21805.3	21871.1	21937.0	22003.0	22069.2	22135.5	22201.9	22268.5	22335.2
1.98	22402.0	22469.0	22536.1	22603.3	22670.7	22738.2	22805.9	22873.7	22941.6	23009.7
1.99	23077.9	23146.2	23214.7	23283.3	23352.1	23421.0	23490.0	23559.1	23628.4	23697.8
2.00	23767.4	23837.1	23907.0	23977.0	24047.2	24117.5	24187.9	24258.4	24329.1	24399.9
2.01	24470.9	24542.0	24613.2	24684.6	24756.1	24827.8	24899.6	24971.5	25043.6	25115.8
2.02	25188.1	25260.6	25333.2	25406.0	25478.9	25551.9	25625.1	25698.4	25771.9	25845.5
2.03	25919.2	25993.1	26067.1	26141.3	26215.6	26290.0	26364.6	26439.3	26514.1	26589.1
2.04	26664.2	26739.5	26814.9	26890.5	26966.2	27042.0	27118.0	27194.1	27270.4	27346.8
2.05	27423.3	27500.0	27576.8	27653.8	27730.9	27808.1	27885.4	27962.9	28040.6	28118.4
2.06	28196.3	28274.4	28352.6	28430.9	28509.4	28588.0	28666.8	28745.7	28824.7	28903.9
2.07	28983.2	29062.7	29142.3	29222.1	29302.0	29382.0	29462.2	29542.5	29622.9	29703.5
2.08	29784.2	29865.1	29946.1	30027.2	30108.5	30189.9	30271.5	30353.2	30435.0	30517.0
2.09	30599.1	30681.4	30763.8	30846.4	30929.1	31011.9	31094.9	31178.0	31261.2	31344.6
2.10	31428.1	31511.8	31595.6	31679.6	31763.7	31847.9	31932.3	32016.8	32101.4	32186.2
2.11	32271.1	32356.2	32441.4	32526.8	32612.3	32697.9	32783.6	32869.5	32955.5	33041.7
2.12	33128.0	33214.5	33301.1	33387.8	33474.6	33561.6	33648.7	33736.0	33823.4	33910.9
2.13	33998.6	34086.4	34174.4	34262.5	34350.7	34439.0	34527.5	34616.1	34704.9	34793.8
2.14	34882.8	34971.9	35061.2	35150.6	35240.2	35329.9	35419.7	35509.7	35599.8	35690.0
2.15	35780.3	35870.7	35961.3	36052.0	36142.9	36233.9	36325.0	36416.3	36507.7	36599.2
2.16	36690.9	36782.7	36874.6	36966.6	37058.8	37151.1	37243.5	37336.0	37428.6	37521.4
2.17	37614.3	37707.4	37800.6	37893.9	37987.3	38080.8	38174.4	38268.1	38362.0	38456.0
2.18	38550.2	38644.5	38739.0	38833.6	38928.4	39023.3	39118.4	39213.6	39309.0	39404.6
2.19	39500.3	39596.2	39692.2	39788.3	39884.6	39981.0	40077.6	40174.4	40271.3	40368.4
2.20	40465.6	40563.0	40660.5	40758.2	40856.0	40954.0	41052.2	41150.6	41249.1	41347.8
2.21	41446.6	41545.6	41644.7	41744.0	41843.4	41943.0	42042.8	42142.7	42242.8	42343.1
2.22	42443.5	42544.1	42644.8	42745.7	42846.8	42948.0	43049.4	43150.9	43252.6	43354.5
2.23	43456.5	43558.7	43661.0	43763.5	43866.2	43969.0	44072.0	44175.2	44278.5	44382.0
2.24	44485.7	44589.5	44693.5	44797.6	44901.9	45006.4	45111.0	45215.8	45320.8	45426.0
2.25	45531.3	45636.8	45742.4	45848.2	45954.1	46060.2	46166.5	46273.0	46379.7	46486.5
2.26	46593.5	46700.6	46807.9	46915.4	47023.0	47130.8	47238.8	47347.0	47455.3	47563.8
2.27	47672.5	47781.3	47890.3	47999.5	48108.9	48218.4	48328.1	48438.0	48548.0	48658.2
2.28	48768.6	48879.2	48989.9	49100.8	49211.8	49323.0	49434.4	49546.0	49657.8	49769.7
2.29	49881.8	49994.1	50106.5	50219.1	50331.9	50444.9	50558.0	50671.3	50784.8	50898.5
2.30	51012.3	51126.3	51240.5	51354.8	51469.3	51584.0	51698.9	51814.0	51929.2	52044.6
2.31	52160.2	52276.0	52391.9	52508.0	52624.3	52740.8	52857.4	52974.2	53091.2	53208.4
2.32	53325.8	53443.3	53561.0	53678.9	53797.0	53915.3	54033.7	54152.3	54271.1	54390.1
2.33	54509.2	54628.5	54748.0	54867.7	54987.6	55107.6	55227.8	55348.2	55468.8	55589.6
2.34	55710.5	55831.6	55952.9	56074.4	56196.1	56318.0	56440.0	56562.2	56684.6	56807.2
2.35	56930.0	57053.0	57176.1	57299.4	57422.9	57546.6	57670.5	57794.6	57918.8	58043.2
2.36	58167.8	58292.6	58417.5	58542.6	58668.0	58793.5	58919.2	59045.0	59171.1	59297.4
2.37	59423.8	59550.5	59677.3	59804.3	59931.5	60058.9	60186.5	60314.3	60442.3	60570.5
2.38	60698.8	60827.3	60955.9	61084.7	61213.8	61343.1	61472.5	61602.1	61731.9	61861.9
2.39	61992.0	62122.4	62253.0	62383.7	62514.6	62645.8	62777.1	62908.6	63040.3	63172.2
2.40	63304.3	63436.5	63569.0	63701.6	63834.4	63967.4	64100.5	64233.9	64367.5	64501.2
2.41	64635.2	64769.4	64903.7	65038.2	65173.0	65307.9	65443.0	65578.3	65713.8	65849.5
2.42	65985.4	66121.5	66257.7	66394.2	66530.8	66667.7	66804.7	66941.9	67079.4	67217.0
2.43	67354.8	67492.8	67631.0	67769.4	67907.9	68046.7	68185.7	68324.8	68464.2	68603.7
2.44	68743.5	68883.5	69023.6	69164.0	69304.5	69445.3	69586.2	69727.4	69868.7	70010.3
2.45	70152.0	70294.0	70436.1	70578.4	70721.0	70863.7	71006.6	71149.7	71293.0	71436.5
2.46	71580.2	71724.1	71868.2	72012.5	72157.0	72301.6	72446.5	72591.6	72736.9	72882.4
2.47	73028.1	73174.0	73320.1	73466.4	73612.8	73759.5	73906.4	74053.5	74200.8	74348.3
2.48	74496.0	74643.9	74792.0	74940.3	75088.8	75237.6	75386.5	75535.6	75685.0	75834.5
2.49	75984.2	76134.2	76284.3	76434.7	76585.3	76736.1	76887.1	77038.3	77189.7	77341.3
2.50	77493.1	77645.1	77797.3	77949.7	78102.3	78255.1	78408.1	78561.3	78714.7	78868.4
2.51	79022.2	79176.3	79330.5	79485.0	79639.7	79794.6	79949.7	80105.0	80260.5	80416.3
2.52	80572.2	80728.3	80884.7	81041.2	81198.0	81354.9	81512.1	81669.5	81827.1	81984.9
2.53	82142.9	82301.1	82459.6	82618.2	82777.1	82936.1	83095.4	83254.9	83414.6	83574.5
2.54	83734.6	83894.9	84055.4	84216.2	84377.1	84538.3	84699.6	84861.2	85023.0	85185.0
2.55	85347.2	85509.6	85672.3	85835.2	85998.2	86161.5	86325.0	86488.7	86652.7	86816.8
2.56	86981.2	87145.8	87310.6	87475.6	87640.8	87806.3	87971.9	88137.8	88303.9	88470.2
2.57	88636.7	88803.4	88970.4	89137.6	89304.9	89472.5	89640.3	89808.4	89976.7	90145.1
2.58	90313.8	90482.7	90651.8	90821.2	90990.8	91160.5	91330.5	91500.7	91671.1	91841.8
2.59	92012.6	92183.7	92355.0	92526.6	92698.3	92870.3	93042.5	93214.9	93387.5	93560.3
2.60	93733.4	93906.7	94080.2	94253.9	94427.8	94602.0	94776.3	94950.9	95125.7	95300.8
2.61	95476.0	95651.5	95827.2	96003.1	96179.3	96355.6	96532.2	96709.0	96886.1	97063.3
2.62	97240.8	97418.5	97596.5	97774.7	97953.1	98131.7	98310.6	98489.6	98668.9	98848.5
2.63	99028.2	99208.2	99388.4	99568.8	99749.4	99930.3	100111	100293	100474	100656
2.64	100838	101020	101202	101385	101568	101751	101934	102117	102301	102485
2.65	102669	102854	103038	103223	103409	103594	103780	103966	104152	104338
2.66	104525	104712	104899	105086	105273	105461	105649	105837	106026	106214
2.67	106403	106592	106781	106971	107161	107351	107541	107731	107922	108113
2.68	108304	108495	108687	108879	109071	109263	109456	109648	109841	110035
2.69	110228	110422	110616	110810	111004	111199	111393	111588	111784	111979
2.70	112175	112371	112567	112764	112960	113157	113354	113552	113749	113947
2.71	114145	114343	114542	114741	114940	115139	115339	115538	115738	115939
2.72	116139	116340	116541	116742	116943	117145	117346	117548	117751	117953
2.73	118156	118359	118562	118766	118970	119174	119378	119583	119788	119993
2.74	120198	120403	120609	120815	121021	121228	121434	121641	121848	122055
2.75	122263	122471	122679	122887	123096	123305	123514	123723	123933	124143
2.76	124353	124563	124773	124984	125195	125406	125617	125829	126041	126253
2.77	126465	126678	126891	127104	127317	127531	127745	127959	128173	128388
2.78	128603	128818	129033	129249	129465	129681	129897	130114	130331	130548
2.79	130765	130983	131200	131419	131637	131855	132074	132293	132513	132732
2.80	132952	133172	133392	133613	133834	134055	134276	134498	134720	134942
2.81	135164	135387	135609	135832	136056	136279	136503	136727	136952	137176
2.82	137401	137626	137851	138077	138303	138529	138755	138982	139209	139436
2.83	139663	139890	140118	140346	140574	140803	141032	141261	141490	141719
2.84	141949	142179	142409	142640	142870	143101	143333	143564	143796	144028
2.85	144260	144493	144725	144958	145192	145425	145659	145893	146128	146362
2.86	146597	146832	147068	147304	147540	147776	148012	148249	148486	148723
2.87	148961	149199	149437	149675	149913	150152	150391	150630	150869	151109
2.88	151349	151589	151830	152070	152312	152553	152794	153036	153278	153520
2.89	153763	154006	154249	154493	154736	154980	155224	155469	155714	155959
2.90	156204	156450	156695	156941	157188	157434	157681	157928	158176	158423
2.91	158671	158919	159168	159416	159665	159914	160164	160413	160663	160914
2.92	161164	161415	161666	161917	162169	162421	162673	162925	163178	163431
2.93	163684	163937	164191	164445	164699	164954	165208	165463	165719	165974
2.91	166230	166486	166742	166999	167256	167513	167770	168028	168285	168544
2.95	168802	169061	169320	169579	169839	170099	170359	170619	170880	171141
2.96	171402	171663	171925	172187	172449	172712	172974	173237	173501	173764
2.97	174028	174292	174557	174821	175086	175352	175617	175883	176149	176415
2.98	176682	176949	177216	177484	177752	178020	178288	178557	178825	179095
2.99	179364	179634	179904	180174	180444	180715	180986	181257	181529	181801
3.00	182073	182345	182618	182891	183164	183438	183712	183986	184260	184535
3.01	184810	185085	185361	185636	185912	186189	186465	186742	187019	187296
3.02	187574	187852	188130	188409	188687	188967	189246	189525	189805	190086
3.03	190366	190647	190928	191209	191491	191773	192055	192338	192621	192904
3.04	193187	193471	193755	194039	194324	194608	194894	195179	195465	195751
3.05	196037	196323	196610	196897	197184	197472	197760	198048	198336	198625
3.06	198914	199203	199492	199783	200073	200363	200654	200945	201237	201528
3.07	201820	202112	202405	202697	202991	203284	203578	203871	204166	204460
3.08	204755	205050	205346	205641	205937	206233	206530	206827	207124	207421
3.09	207719	208017	208315	208614	208912	209221	209511	209810	210110	210411
3.10	210711	211012	211313	211614	211916	212218	212520	212823	213125	213429
3.11	213732	214036	214340	214644	214949	215254	215559	215865	216171	216477
3.12	216783	217090	217397	217704	218012	218320	218628	218937	219246	219555
3.13	219864	220174	220484	220794	221105	221416	221727	222039	222350	222663
3.14	222975	223288	223601	223914	224227	224541	224855	225170	225485	225800
3.15	226115	226431	226747	227063	227379	227696	228013	228331	228649	228967
3.16	229285	229604	229922	230242	230561	230881	231201	231521	231842	232163
3.17	232484	232806	233128	233450	233772	234095	234418	234742	235066	235390
3.18	235714	236039	236364	236689	237014	237340	237666	237993	238320	238647
3.19	238974	239302	239630	239958	240287	240616	240946	241275	241605	241935
3.20	242266	242597	242928	243259	243591	243923	244255	244588	244920	245254
3.21	245587	245920	246255	246589	246924	247259	247595	247930	248266	248602
3.22	248939	249276	249613	249951	250289	250627	250965	251304	251643	251982
3.23	252322	252662	253002	253343	253684	254025	254367	254709	255051	255393
3.24	255736	256079	256423	256767	257111	257455	257800	258145	258490	258836
3.25	259182	259528	259875	260222	260569	260916	261264	261612	261960	262309
3.26	262658	263007	263357	263707	264057	264408	264759	265110	265462	265814
3.27	266166	266519	266871	267225	267578	267932	268286	268641	268995	269351
3.28	269706	270062	270418	270774	271131	271488	271845	272203	272561	272919
3.29	273278	273637	273996	274355	274715	275075	275435	275796	276157	276518
3.30	276880	277242	277605	277967	278331	278694	279058	279422	279786	280151
3.31	280516	280881	281247	281613	281979	282346	282712	283080	283447	283815
3.32	284183	284552	284920	285290	285659	286029	286399	286770	287140	287512
3.33	287883	288255	288627	288999	289372	289745	290118	290492	290866	291240
3.34	291615	291990	292365	292741	293117	293493	293870	294247	294624	295002
3.35	295380	295758	296137	296516	296895	297275	297655	298035	298416	298797
3.36	299178	299560	299941	300324	300706	301089	301472	301856	302239	302624
3.37	303008	303393	303778	304163	304549	304935	305322	305709	306096	306483
3.38	306871	307259	307648	308037	308426	308815	309205	309595	309986	310377
3.39	310768	311159	311551	311943	312335	312728	313121	313515	313908	314302
3.40	314697	315092	315487	315882	316278	316674	317070	317467	317864	318261
3.41	318659	319057	319455	319854	320253	320652	321052	321452	321852	322253
3.42	322654	323055	323457	323859	324262	324665	325068	325471	325875	326279
3.43	326684	327089	327493	327899	328305	328711	329118	329525	329932	330339
3.44	330747	331155	331564	331973	332382	332792	333202	333612	334023	334434
3.45	334845	335257	335668	336081	336493	336906	337319	337733	338147	338561
3.46	338976	339391	339806	340222	340638	341054	341471	341888	342305	342723
3.47	343141	343559	343978	344397	344817	345237	345657	346077	346498	346919
3.48	347341	347763	348185	348608	349030	349454	349877	350301	350725	351150
3.49	351575	352000	352426	352852	353278	353705	354132	354560	354987	355416
3.50	355844	356273	356702	357131	357561	357991	358422	358852	359284	359715
3.51	360147	360579	361012	361445	361878	362312	362746	363180	363615	364050
3.52	364485	364921	365357	365794	366231	366668	367106	367544	367982	368421
3.53	368860	369299	369739	370179	370619	371060	371501	371943	372384	372826
3.54	373269	373712	374155	374599	375043	375487	375932	376377	376822	377268
3.55	377714	378160	378607	379054	379502	379950	380398	380846	381295	381744
3.56	382194	382644	383094	383545	383996	384447	384899	385351	385804	386257
3.57	386710	387164	387617	388072	388526	388982	389437	389893	390349	390805
3.58	391262	391719	392177	392634	393092	393551	394010	394469	394929	395389
3.59	395849	396310	396770	397232	397693	398155	398618	399081	399544	400007
3.60	400471	400935	401400	401865	402330	402796	403262	403728	404195	404662
3.61	405130	405598	406066	406535	407004	407473	407943	408413	408883	409354
3.62	409825	410296	410768	411240	411713	412186	412659	413133	413607	414081
3.63	414556	415031	415507	415983	416459	416935	417412	417890	418367	418846
3.64	419324	419803	420282	420761	421241	421721	422202	422683	423164	423646
3.65	424128	424610	425093	425576	426060	426543	427028	427512	427997	428482
3.66	428968	429454	429941	430427	430915	431402	431890	432379	432867	433356
3.67	433846	434336	434826	435316	435807	436298	436790	437282	437774	438267
3.68	438760	439254	439748	440242	440737	441232	441727	442223	442719	443216
3.69	443713	444210	444708	445206	445704	446203	446702	447201	447701	448201
3.70	448702	449203	449704	450206	450708	451211	451714	452217	452721	453225
3.71	453729	454234	454739	455244	455750	456257	456763	457270	457778	458286
3.72	458794	459303	459812	460321	460831	461341	461851	462362	462873	463385
3.73	463897	464409	464922	465435	465949	466463	466977	467492	468007	468522
3.74	469038	469554	470071	470588	471105	471623	472141	472660	473179	473698
3.75	474218	474738	475258	475779	476300	476822	477344	477866	478389	478912
3.76	479435	479959	480483	481008	481533	482058	482584	483110	483637	484164
3.77	484691	485219	485747	486275	486804	487333	487863	488393	488923	489454
3.78	489985	490516	491048	491580	492113	492646	493180	493713	494247	494782
3.79	495317	495852	496388	496924	497461	497998	498535	499073	499611	500149
3.80	500688	501227	501767	502307	502847	503388	503929	504471	505013	505555
3.81	506098	506641	507185	507729	508273	508818	509363	509908	510454	511000
3.82	511547	512094	512642	513190	513738	514287	514836	515385	515935	516485
3.83	517036	517587	518139	518690	519243	519795	520348	520901	521455	522009
3.84	522564	523119	523674	524230	524786	525343	525900	526457	527015	527573
3.85	528132	528691	529250	529810	530370	530930	531491	532053	532614	533176
3.86	533739	534302	534865	535429	535993	536558	537123	537688	538254	538820
3.87	539387	539954	540521	541089	541657	542226	542795	543364	543934	544504
3.88	545075	545646	546218	546790	547362	547935	548508	549082	549656	550230
3.89	550805	551380	551956	552531	553108	553684	554262	554839	555417	555995
3.90	556574	557153	557732	558312	558893	559473	560054	560636	561218	561800
3.91	562383	562966	563550	564133	564718	565303	565889	566474	567060	567647
3.92	568234	568821	569409	569997	570586	571175	571764	572354	572944	573535
3 93	574126	574717	575309	575902	576494	577087	577681	578275	578869	579464
3.94	580059	580655	581251	581847	582444	583041	583639	584237	584836	585435
3.95	586934	586634	587234	587835	588436	589037	589639	590241	590844	591447
3.96	592051	592655	593259	593864	594470	595075	595681	596288	596895	597502
3.97	598110	598718	599327	599936	600545	601155	601765	602376	602987	603598
3.98	604210	604822	605435	606048	606662	607276	607890	608505	609120	609736
3.99	610352	610969	611586	612203	612821	613439	614058	614677	615297	615917
4.00	616537	617158	617779	618401	619023	619645	620268	620891	621515	622139
4.01	622764	623389	624014	624640	625266	625893	626520	627148	627776	628404
4 02	629033	629662	630292	630922	631553	632184	632815	633447	634079	634712
4.03	635345	635979	636613	637247	637882	638517	639153	639789	640426	641063
4.04	641700	642338	642976	643615	644254	644894	645534	646174	646815	647457
4.05	648999	648741	649384	650027	650671	651315	651959	652604	653249	653895
4.06	654541	655188	655836	656482	657130	657778	658427	659076	659726	660376
4.07	661026	661677	662328	662980	663632	664284	664938	665591	666245	666899
4.08	667554	668209	668865	669521	670177	670834	671491	672149	672807	673466
4.09	674125	674784	675444	676105	676766	677427	678089	678751	679413	680076
4.10	680740	681404	682068	682733	683398	684064	684730	685397	686064	686731
4.11	687399	688067	688736	689405	690075	690745	691416	692087	692759	693431
4.12	694103	694776	695449	696123	696797	697471	698146	698822	699498	700174
4.13	700851	701528	702206	702884	703562	704241	704921	705601	706281	706962
4.14	707643	708325	709007	709689	710372	711055	711739	712423	713108	713793
4.15	714479	715165	715852	716538	717226	717914	718602	719291	719980	720670
4.16	721360	722050	722741	723433	724124	724817	725509	726203	726896	727590
4.17	728285	728980	729675	730371	731067	731764	732461	733159	733857	734556
4.18	735255	735954	736654	747354	738055	738756	739458	740160	740863	741566
4.19	742269	742973	743677	744382	745087	745793	746499	747205	747912	748620
4.20	749328	750036	750745	751454	752164	752874	753584	754295	755007	755719
4.21	756431	757144	757857	758571	759285	759999	760714	761430	762146	762862
4.22	763579	764296	765014	765732	766451	767170	767889	768609	769330	770051
4.23	770772	771494	772216	772938	773662	774385	775109	775834	776559	777284
4.24	778010	778736	779463	780190	780918	781646	782375	783104	783833	784563
4.25	785294	786025	786756	787488	788220	788953	789686	790419	791153	791888
4.26	792623	793358	794094	794831	795568	796305	797043	707781	798520	799259
4.27	799999	800739	801480	802221	802962	803705	804447	805190	805934	806678
4.28	807422	808167	808912	809658	810405	811152	811899	812647	813395	814144
4.29	814893	815643	816393	817143	817894	818646	819398	820151	820904	821657
4.30	822411	823166	823921	824676	825432	826188	826945	827703	828461	829219
4.31	829978	830737	831497	832257	833018	833779	834541	835303	836065	836828
4.32	837592	838356	839121	839886	840651	841417	842184	842951	843718	844486
4.33	845255	846024	846793	847563	848334	849104	849876	850648	851420	852193
4.34	852966	853740	854514	855289	856064	856839	857616	858392	859169	859947
4.35	860725	861504	862283	863062	863842	864623	865404	866185	866967	867750
4.36	868533	869317	870101	870885	871670	872455	873241	874028	874815	875602
4.37	876390	877178	877967	878757	879546	880337	881128	881919	882711	883503
4.38	884296	885089	885883	886678	887472	888268	889064	889860	890657	891454
4.39	892252	893050	893849	894649	895448	896249	897050	897851	898653	899455
4.40	900258	901061	901865	902669	903474	904279	905085	905891	906698	907505
4.41	908313	909121	909930	910739	911549	912359	913170	913981	914793	915605
4.42	916418	917231	918045	918859	919674	920489	921305	922121	922938	923755
4.43	924573	925391	926210	927029	927849	928669	929490	930311	931133	931955
4.44	932778	933601	934425	935249	936074	936899	937725	938551	939378	940205
4.45	941033	941861	942690	943519	944349	945179	946010	946841	947673	948505
4.46	949338	950171	951005	951839	952674	953509	954345	955181	956018	956855
4.47	957693	958531	959370	960209	961049	961890	962730	963572	964414	965256
4.48	966099	966942	967786	968631	969476	970321	971167	972013	972860	973708
4.49	974556	975405	976254	977104	977954	978804	979656	980508	981360	982213
4.50	983066	983920	984774	985629	986485	987340	988197	989054	989911	990769
4.51	991628	992487	993346	994206	995066	995927	996788	997650	998513	999376
4.52	1000239	1001103	1001968	1002833	1003699	1004565	1005432	1006300	1007168	1008036
4.53	1008905	1009774	1010644	1011514	1012385	1013256	1014128	1015001	1015874	1016747
4.54	1017621	1018496	1019371	1020246	1021122	1021999	1022876	1023754	1024632	1025511
4.55	1026390	1027270	1028150	1029031	1029913	1030795	1031677	1032561	1033444	1034328
4.56	1035213	1036098	1036984	1037870	1038756	1039643	1040531	1041419	1042308	1043197
4.57	1044087	1044977	1045868	1046759	1047651	1048544	1049437	1050330	1051224	1052119
4.58	1053014	1053910	1054806	1055703	1056600	1057498	1058396	1059295	1060194	1061094
4.59	1061995	1062896	1063797	1064700	1065602	1066505	1067409	1068313	1069218	1070123
4.60	1071029	1071935	1072842	1073749	1074656	1075565	1076474	1077383	1078293	1079203
4.61	1080114	1081026	1081938	1082850	1083763	1084677	1085591	1086506	1087422	1088338
4.62	1089254	1090171	1091089	1092007	1092925	1093845	1094764	1095685	1096606	1097527
4.63	1098449	1099372	1100295	1101218	1102142	1103067	1103992	1104918	1105845	1106772
4.64	1107699	1108627	1109555	1110484	1111414	1112344	1113274	1114205	1115137	1116069
4.65	1117002	1117935	1118869	1119803	1120738	1121674	1122610	1123546	1124483	1125421
4.66	1126359	1127298	1128237	1129177	1130118	1131059	1132000	1132942	1133885	1134828
4.67	1135772	1136716	1137661	1138606	1139552	1140499	1141446	1142393	1143341	1144290
4.68	1145239	1146189	1147139	1148090	1149041	1149993	1150945	1151899	1152852	1153806
4.69	1154761	1155716	1156672	1157629	1158585	1159543	1160501	1161460	1162419	1163379
4.70	1164339	1165300	1166261	1167223	1168186	1169149	1170112	1171076	1172041	1173006
4.71	1173972	1174938	1175905	1176873	1177841	1178810	1179779	1180749	1181719	1182690
4.72	1183662	1184634	1185606	1186580	1187553	1188527	1189502	1190478	1191453	1192430
4.73	1193407	1194385	1195363	1196342	1197321	1198301	1199281	1200262	1201244	1202226
4.74	1203209	1204192	1205176	1206160	1207145	1208130	1209116	1210103	1211090	1212078
4.75	1213066	1214055	1215044	1216035	1217025	1218016	1219008	1220001	1220993	1221987
4.76	1222981	1223976	1224971	1225967	1226964	1227961	1228959	1229957	1230956	1231955
4.77	1232955	1233955	1234956	1235957	1236959	1237962	1238965	1239969	1240973	1241978
4.78	1242983	1243989	1244996	1246003	1247011	1248019	1249028	1250037	1251047	1252058
4.79	1253069	1254081	1255093	1256106	1257119	1258133	1259148	1260163	1261179	1262195
4.80	1263212	1264230	1265248	1266266	1267286	1268306	1269326	1270347	1271369	1272391
4.81	1273414	1274437	1275461	1276486	1277511	1278536	1279562	1280589	1281616	1282644
4.82	1283673	1284702	1285732	1286762	1287793	1288825	1289857	1290889	1291923	1292957
4.83	1293991	1295026	1296062	1297098	1298134	1299172	1300210	1301248	1302287	1303327
4.84	1304367	1305408	1306449	1307491	1308534	1309577	1310621	1311665	1312710	1313756
4.85	1314802	1315849	1316896	1317944	1318993	1320042	1321092	1322142	1323193	1324245
4.86	1325297	1326350	1327403	1328457	1329511	1330566	1331622	1332678	1333735	1334792
4.87	1335850	1336908	1337968	1339027	1340088	1341148	1342210	1343272	1344335	1345398
4.88	1346462	1347527	1348592	1349658	1350724	1351791	1352859	1353927	1354996	1356066
4.89	1357136	1358207	1359278	1360350	1361422	1362495	1363569	1364643	1365718	1366794
4.90	1367870	1368947	1370024	1371101	1372180	1373258	1374338	1375418	1376499	1377580
4.91	1378662	1379745	1380828	1381912	1382996	1384081	1385167	1386253	1387340	1388428
4.92	1389516	1390605	1391694	1392783	1393874	1394965	1396056	1397149	1398241	1399335
4.93	1400429	1401524	1402619	1403715	1404812	1405909	1407007	1408105	1409204	1410304
4.94	1411404	1412505	1413606	1414708	1415810	1416913	1418017	1419121	1420226	1421332
4.95	1422438	1423545	1424652	1425760	1426869	1427978	1429088	1430198	1431309	1432421
4.96	1433533	1434646	1435760	1436874	1437988	1439104	1440220	1441336	1442454	1443572
4.97	1444690	1445809	1446929	1448050	1449171	1450293	1451415	1452538	1453662	1454786
4.98	1455911	1457036	1458162	1459288	1460415	1461543	1462671	1463800	1464930	1466060
4.99	1467191	1468323	1469455	1470588	1471721	1472855	1473990	1475125	1476261	1477398
5.00	1478535	1479673	1480811	1481950	1483089	1484230	1485370	1486512	1487654	1488797
5.01	1489940	1491084	1492229	1493374	1494520	1495667	1496814	1497962	1499110	1500259
5.02	1501409	1502559	1503710	1504862	1506014	1507167	1508320	1509474	1510629	1511784
5.03	1512940	1514097	1515254	1516412	1517570	1518730	1519889	1521050	1522211	1523373
5.04	1524535	1525698	1526861	1528025	1529190	1530356	1531522	1532688	1533856	1535023
5.05	1536192	1537361	1538531	1539701	1540872	1542044	1543216	1544389	1545563	1546737
5.06	1547912	1549088	1550264	1551441	1552619	1553797	1554976	1556155	1557336	1558516
5.07	1559698	1560880	1562062	1563246	1564429	1565614	1566799	1567985	1569171	1570358
5.08	1571546	1572734	1573923	1575113	1576303	1577494	1578685	1579878	1581070	1582264
5.09	1583458	1584653	1585849	1587045	1588242	1589439	1590638	1591836	1593036	1594236
5.10	1595437	1596638	1597841	1599043	1600247	1601451	1602656	1603861	1605067	1606274
5.11	1607481	1608689	1609897	1611107	1612316	1613527	1614738	1615950	1617162	1618375
5.12	1619589	1620803	1622018	1623234	1624450	1625667	1626884	1628102	1629321	1630541
5.13	1631761	1632982	1634204	1635426	1636649	1637872	1639096	1640321	1641547	1642773
5.14	1644000	1645228	1646456	1647685	1648914	1650144	1651375	1652606	1653839	1655071
5.15	1656305	1657539	1658773	1660008	1661244	1662481	1663718	1664956	1666194	1667433
5.16	1668673	1669913	1671155	1672396	1673639	1674882	1676126	1677370	1678616	1679862
5.17	1681108	1682355	1683603	1684852	1686102	1687352	1688602	1689854	1691106	1692359
5.18	1693612	1694866	1696120	1697375	1698631	1699888	1701145	1702403	1703661	1704920
5.19	1706180	1707441	1708702	1709964	1711228	1712490	1713754	1715019	1716284	1717550
5.20	1718817	1720084	1721352	1722621	1723891	1725161	1726431	1727703	1728975	1730248
5.21	1731521	1732795	1734069	1735345	1736620	1737897	1739174	1740452	1741731	1743010
5.22	1744290	………..	………..	………..	………..	………..	………..	………..	………..	………..

**Table II:** 1958 *He*^4^ vapor pressure-temperature scale, *T* in °K as a function of *P* in millimeters mercury at 0° C and standard gravity, 980.665 cm/sec^2^

*P*	0		1		2		3		4		5		6		7		8		9	
																				
0.01	0.7907	65	7972	61	8033	57	8090	53	8143	50	8193	47	8240	45	8285	43	8328	40	8368	39
.02	.8407	38	8445	35	8480	35	8515	33	8548	32	8580	32	8612	30	8642	29	8671	28	8699	28
.03	.8727	27	8754	26	8780	25	8805	25	8830	24	8854	24	8878	23	8901	22	8923	22	8945	22
.04	.8967	21	8988	21	9009	20	9029	20	9049	19	9068	20	9088	18	9106	19	9125	18	9143	18
.05	.9161	18	9179	17	9196	17	9213	17	9230	16	9246	17	9263	16	9279	15	9294	16	9310	15
.06	.9325	15	9340	15	9355	15	9370	15	9385	14	9399	14	9413	14	9427	14	9441	14	9455	13
.07	.9468	14	9482	13	9495	13	9508	13	9521	13	9534	12	9546	13	9559	12	9571	12	9583	12
.08	.9595	12	9607	12	9619	12	9631	12	9643	11	9654	11	9665	12	9677	11	9688	11	9699	11
.09	.9710	11	9721	11	9732	10	9742	11	9753	10	9763	11	9774	10	9784	10	9794	10	9804	10
.10	.9814	10	9824	10	9834	10	9844	10	9854	9	9863	10	9873	10	9883	9	9892	9	9901	10
.11	.9911	9	9920	9	9929	9	9938	9	9947	9	9956	9	9965	9	9974	9	9983	8	9991	9
.12	1.0000	9	0009	8	0017	9	0026	8	0034	8	0042	9	0051	8	0059	8	0067	8	0075	8
.13	1.0083	8	0091	8	0099	8	0107	8	0115	8	0123	8	0131	8	0139	7	0146	8	0154	8
.14	1.0162	7	0169	8	0177	7	0184	8	0192	7	0199	7	0206	8	0214	7	0221	7	0228	8
.15	1.0236	7	0243	7	0250	7	0257	7	0264	7	0271	7	0278	7	0285	7	0292	7	0299	6
.16	1.0305	7	0312	7	0319	7	0326	6	0332	7	0339	7	0346	6	0352	7	0359	6	0365	7
.17	1.0372	6	0378	7	0385	6	0391	7	0398	6	0404	6	0410	7	0417	6	0423	6	0429	6
.18	1.0435	6	0441	7	0448	6	0454	6	0460	6	0466	6	0472	6	0478	6	0484	6	0490	6
.19	1.0496	6	0502	6	0508	5	0513	6	0519	6	0525	6	0531	6	0537	5	0542	6	0548	6
0.2	1.0554	55	0609	54	0663	52	0715	49	0764	49	0813	46	0859	45	0904	44	0948	43	0991	41
.3	1.1032	41	1073	39	1112	38	1150	38	1188	36	1224	36	1260	35	1295	34	1329	33	1362	33
.4	1.1395	32	1427	32	1459	31	1490	30	1520	30	1550	29	1579	29	1608	28	1636	27	1663	28
.5	1.1691	27	1718	26	1744	26	1770	26	1796	25	1821	25	1846	25	1871	24	1895	24	1919	23
.6	1.1942	24	1966	23	1989	22	2011	23	2034	22	2056	22	2078	21	2099	21	2120	21	2141	21
.7	1.2162	21	2183	20	2203	20	2223	20	2243	20	2263	20	2283	19	2302	19	2321	19	2340	19
.8	1.2359	18	2377	18	2395	19	2414	18	2432	17	2449	18	2467	IS	2485	17	2502	17	2519	17
.9	1.2536	17	2553	17	2570	16	2586	17	2603	16	2619	16	2635	16	2651	16	2667	16	2683	16
1.0	1.2699	15	2714	16	2730	15	2745	15	2760	15	2775	15	2790	15	2805	15	2820	14	2834	15
1.1	1.2849	14	2863	15	2878	14	2892	14	2906	14	2920	14	2934	14	2948	13	2961	14	2975	14
1.2	1.2989	13	3002	13	3015	14	3029	13	3042	13	3055	13	3068	13	3081	13	3094	13	3107	12
1.3	1.3119	13	3132	13	3145	12	3157	12	3169	13	3182	12	3194	12	3206	12	3218	12	3230	12
1.4	1.3242	12	3254	12	3266	12	3278	12	3290	11	3301	12	3313	12	3325	11	3336	12	3348	11
1.5	1.3359	11	3370	11	3381	12	3393	11	3404	11	3415	11	3426	11	3437	11	3448	11	3459	10
1.6	1.3469	11	3480	11	3491	10	3501	11	3512	11	3523	10	3533	11	3544	10	3554	10	3564	11
1.7	1.3575	10	3585	10	3595	10	3605	10	3615	10	3625	10	3635	10	3645	10	3655	10	3665	10
1.8	1.3675	10	3685	10	3695	9	3704	10	3714	10	3724	9	3733	10	3743	9	3752	10	3762	9
1.9	1.3771	9	3780	10	3790	9	3799	10	3809	9	3818	9	3827	9	3836	9	3845	9	3854	9
2	1.3863	89	3952	86	4038	82	4120	80	4200	77	4277	75	4352	73	4425	71	4496	69	4565	67
3	1.4632	65	4697	63	4760	63	4823	60	4883	60	4943	58	5001	56	5057	56	5113	55	5168	53
4	1.5221	.53	5274	51	5325	51	5376	49	5425	49	5474	48	5522	47	5569	47	5616	46	5662	45
5	1.5707	44	5751	44	5795	43	5838	42	5880	42	5922	41	5963	41	6004	40	6044	40	6084	39
6	1.6123	39	6162	38	6200	38	6238	37	6275	37	6312	36	6348	36	6384	36	6420	35	6455	35
7	1.6490	35	6525	34	6559	34	6593	33	6626	33	6659	33	6692	32	6724	32	6756	32	6788	32
8	1.6820	31	6851	31	6882	31	6913	30	6943	30	6973	30	7003	30	7033	29	7062	29	7091	29
9	1.7120	28	7148	29	7177	28	7205	28	7233	28	7261	27	7288	28	7316	27	7343	27	7370	26
10	1.7396	27	7423	26	7449	26	7475	26	7501	26	7527	25	7552	26	7578	25	7603	25	7628	25
11	1.7653	25	7678	24	7702	25	7727	24	7751	24	7775	24	7799	24	7823	23	7846	24	7870	23
12	1.7893	23	7916	23	7939	23	7962	23	7985	23	8008	22	8030	23	8053	22	8075	22	8 097	22
13	1.8119	22	8141	22	8163	21	8184	22	8206	21	8227	22	8249	21	8270	21	8291	21	8312	21
14	1.8333	20	8353	21	8374	21	8395	20	8415	20	8435	21	8456	20	8476	20	8496	20	8516	20
15	1.8536	19	8555	20	8575	20	8595	19	8614	20	8634	19	8653	19	8672	19	8691	19	8710	19
16	1.8729	19	8748	19	8767	18	8785	19	8804	19	8823	18	8841	19	8860	18	8878	18	8896	18
17	1.8914	18	8932	18	8950	18	8968	18	8986	18	9004	18	9022	17	9039	18	9057	17	9074	18
18	1.9092	17	9109	17	9126	18	9144	17	9161	17	9178	17	9195	17	9212	17	9229	17	9246	16
19	1.9262	17	9279	17	9296	16	9312	17	9329	16	9345	17	9362	16	9378	16	9394	17	9411	16
20	1.9427	16	9443	16	9459	16	9475	16	9491	16	9507	16	9523	16	9539	15	9554	16	9570	16
21	1.9586	16	9602	15	9617	15	9632	16	9648	15	9663	16	9679	15	9694	15	9709	15	9724	16
22	1.9740	15	9755	15	9770	15	9785	15	9800	15	9815	15	9830	14	9844	15	9859	15	9874	15
23	1.9889	14	9903	15	9918	14	9932	15	9947	14	9961	15	9976	14	9990	15	0005	14	0019	14
24	2.0033	15	0048	14	0062	14	0076	14	0090	14	0104	14	0118	14	0132	14	0146	14	0160	14
25	2.0174	14	0188	14	0202	13	0215	14	0229	14	0243	14	0257	13	0270	14	0284	13	0297	14
26	2.0311	13	0324	14	0338	13	0351	14	0365	13	0378	13	0391	14	0405	13	0418	13	0431	13
27	2.0444	14	0458	13	0471	13	0484	13	0497	13	0510	13	0523	13	0536	13	0549	13	0562	13
28	2.0575	13	0588	12	0600	13	0613	13	0626	13	0639	13	0652	12	0664	13	0677	13	0690	12
29	2.0702	13	0715	12	0727	13	0740	12	0752	13	0765	12	0777	13	0790	12	0802	12	0814	13
30	2.0827	12	0839	12	0851	12	0863	13	0876	12	0888	12	0900	12	0912	12	0924	12	0936	13
31	2.0949	12	0961	12	0973	12	0985	12	0997	12	1009	12	1021	11	1032	12	1044	12	1056	12
32	2.1068	12	1080	12	1092	11	1103	12	1115	12	1127	12	1139	11	1150	12	1162	12	1174	11
33	2.1185	12	1197	11	1208	12	1220	11	1231	12	1243	11	1254	12	1266	11	1277	12	1289	11
34	2.1300	12	1312	11	1323	11	1334	12	1346	11	1357	11	1368	11	1379	12	1391	11	1402	11
35	2.1413	11	1424	12	1436	11	1447	11	1458	11	1469	11	1480	11	1491	11	1502	11	1513	11
36	2.1524	11	1535	11	1546	11	1557	11	1568	11	1579	11	1590	11	1601	11	1612	11	1623	11
37	2.1634	10	1644	11	1655	11	1666	11	1677	11	1688	10	1698	11	1709	11	1720	11	1731	10
38	2.1741	11	1752	11	1763	10	1773	11	1784	11	1795	10	1805	11	1816	10	1826	11	1837	11
39	2.1848	10	1858	11	1869	11	1880	10	1890	10	1900	10	1910	11	1921	10	1931	11	1942	10
40	2.1952	10	1962	11	1973	10	1983	10	1993	11	2004	10	2014	10	2024	10	2034	10	2044	11
41	2.2055	10	2065	10	2075	10	2085	10	2095	10	2105	10	2115	11	2126	10	2136	10	2146	10
42	2.2156	10	2166	10	2176	10	2186	10	2196	10	2206	10	2216	9	2225	10	2235	10	2245	10
43	2.2255	10	2265	10	2275	10	2285	9	2294	10	2304	10	2314	10	2324	10	2334	9	2343	10
44	2.2353	10	2363	9	2372	10	2382	10	2392	9	2401	10	2411	10	2421	9	2430	10	2440	10
45	2.2450	9	2459	9	2468	10	2478	10	2488	10	2498	9	2507	9	2516	9	2525	10	2535	9
46	2.2544	10	2554	9	2563	10	2573	9	2582	9	2591	10	2601	9	2610	9	2619	10	2629	9
47	2.2638	9	2647	9	2656	10	2666	9	2675	9	2684	9	2693	10	2703	9	2712	9	2721	9
48	2.2730	9	2739	9	2748	9	2757	10	2767	9	2776	9	2785	9	2794	9	2803	9	2812	9
49	2.2821	9	2830	9	2839	9	2848	9	2857	9	2866	9	2875	9	2884	9	2893	9	2902	9
50	2.2911	88	2999	87	3086	86	3172	85	3257	84	3341	83	3424	82	3506	81	3587	79	3666	79
60	2.3745	78	3823	78	3901	76	3977	75	4052	75	4127	74	4201	73	4274	73	4347	71	4418	71
70	2.4489	71	4560	69	4629	69	4698	68	4766	68	4834	67	4901	66	4967	66	5033	66	5099	64
80	2.5163	64	5227	64	5291	63	5354	63	5417	62	5479	61	5540	61	5601	61	5662	60	5722	59
90	2.5781	60	5841	58	5899	59	5958	57	6015	58	6073	57	6130	56	6186	57	6243	55	6298	56
100	2.6354	55	6409	55	6464	54	6518	54	6572	53	6625	54	6679	53	6732	52	6784	52	6836	52
110	2.6888	52	6940	51	6991	51	7042	51	7093	50	7143	50	7193	50	7243	49	7292	49	7341	49
120	2.7390	49	7439	48	7487	48	7535	48	7583	48	7631	47	7678	47	7725	47	7772	46	7818	47
130	2.7865	46	7911	46	7957	45	8002	46	8048	45	8093	45	8138	44	8182	45	8227	44	8271	44
140	2.8315	44	8459	43	8402	44	8446	43	8489	43	8532	43	8575	42	8617	42	8659	43	8702	42
150	2.8744	41	8785	42	8827	42	8869	41	8910	41	8951	41	8992	40	9032	41	9073	40	9113	40
160	2.9153	40	9193	40	9233	40	9273	39	9312	40	9352	39	9391	39	9430	39	9469	39	9508	38
170	2.9546	39	9.585	38	9623	38	9661	38	9699	38	9737	37	9774	38	9812	38	9850	36	9886	38
180	2.9924	37	9961	36	9997	37	0034	37	0071	36	0107	36	0143	36	0179	36	0215	36	0251	36
190	3.0287	36	0323	35	0358	35	0393	36	0429	35	0464	35	0499	35	0534	34	0568	35	0603	34
200	3.0637	35	0672	34	0706	34	0740	34	0774	34	0808	34	0842	34	0876	33	0909	34	0943	33
210	3.0976	34	1010	33	1043	33	1076	33	1109	33	1142	32	1174	33	1207	33	1240	32	1272	32
220	3.1304	33	1337	32	1369	32	1401	32	1433	32	1465	31	1496	32	1528	32	1560	31	1591	31
230	3.1622	32	1654	31	1685	31	1716	31	1747	31	1778	31	1809	31	1840	30	1870	31	1901	30
240	3.1931	31	1962	30	1992	30	2022	30	2052	30	2082	30	2112	30	2142	30	2172	30	2202	29
250	3.2231	30	2261	30	2291	29	2320	29	2349	30	2379	29	2408	29	2437	29	2466	29	2495	29
260	3.2524	28	2552	29	2581	29	2610	28	2638	29	2667	28	2695	29	2724	28	2752	28	2780	28
270	3.2808	28	2836	28	2864	28	2892	28	2920	28	2948	28	2976	27	3003	28	3031	27	3058	28
280	3.3086	27	3113	28	3141	27	3168	27	3195	27	3222	27	3249	27	3276	27	3303	27	3330	27
290	3.3357	27	3384	26	3410	27	3437	26	3463	27	3490	26	3516	27	3543	26	3569	26	3595	27
300	3.3622	26	3648	26	3674	26	3700	26	3726	26	3752	26	3778	25	3803	26	3829	26	3855	25
310	3.3880	26	3906	25	3931	26	3957	25	3982	26	4008	25	4033	25	4058	25	4083	26	4109	25
320	3.4134	25	4159	25	4184	25	4209	24	4233	25	4258	25	4283	25	4308	24	4332	25	4357	25
330	3.4382	24	4406	25	4431	24	4455	24	4479	25	4504	24	4528	24	4552	24	4576	25	4601	24
340	3.4625	24	4649	24	4673	24	4697	24	4721	23	4744	24	4768	24	4792	24	4816	23	4839	24
350	3.4863	23	4886	24	4910	23	4933	24	4957	23	4980	24	5004	23	5027	23	5050	23	5073	24
360	3.5097	23	5120	23	5143	23	5166	23	5189	23	5212	23	5235	23	5258	22	5280	23	5303	23
370	3.5326	23	5349	22	5371	23	5394	23	5417	22	5439	23	5462	22	5484	22	5506	23	5529	22
380	3.5551	22	5573	23	5596	22	5618	22	5640	22	5662	22	5684	22	5706	22	5728	22	5750	22
390	3.5772	22	5794	22	5816	22	5838	22	5860	21	5881	22	5903	22	5925	22	5947	21	5968	22
400	3.5990	21	6011	22	6033	21	6054	22	6076	21	6097	22	6119	21	6140	21	6161	21	6182	22
410	3.6204	21	6225	21	6246	21	6267	21	6288	21	6309	21	6330	21	6351	21	6372	21	6393	21
420	3.6414	21	6435	21	6456	21	6477	20	6497	21	6518	21	6539	20	6559	21	6580	21	6601	20
430	3.6621	21	6642	20	6662	21	6683	20	6703	21	6724	20	6744	20	6764	21	6785	20	6805	20
440	3.6825	20	6845	21	6866	20	6886	20	6906	20	6926	20	6946	20	6966	20	6986	20	7006	20
450	3.7026	20	7046	20	7066	20	7086	19	7105	20	7125	20	7145	20	7165	19	7184	20	7204	20
460	3.7224	19	7243	20	7263	19	7282	20	7302	20	7322	19	7341	19	7360	20	7380	19	7399	20
470	3.7419	19	7438	19	7457	20	7477	19	7496	19	7515	19	7534	19	7553	20	7573	19	7592	19
480	3.7611	19	7630	19	7649	19	7668	19	7687	19	7706	19	7725	19	7744	19	7763	18	7781	19
490	3.7800	19	7819	19	7838	19	7857	18	7875	19	7894	19	7913	18	7931	19	7950	19	7969	18
500	3.7987	19	8006	18	8024	19	8043	18	8061	19	8080	18	8098	19	8117	18	8135	18	8153	19
510	3.8172	18	8190	18	8208	19	8227	18	8245	18	8263	18	8281	18	8299	18	8317	19	8336	18
520	3.8354	18	8372	18	8390	18	8408	18	8426	18	8444	18	8462	18	8480	18	8498	18	8516	17
530	3.8533	18	8551	18	8569	18	8587	18	8605	17	8622	18	8640	18	8658	18	8676	17	8693	18
540	3.8711	17	8728	18	8746	18	8764	17	8781	18	8799	17	8816	18	8834	17	8851	18	8869	17
550	3.8886	17	8903	18	8921	17	8938	17	8955	18	8973	17	8990	17	9007	18	9025	17	9042	17
560	3.9059	17	9076	17	9093	18	9111	17	9128	17	9145	17	9162	17	9179	17	9196	17	9213	17
570	3.9230	17	9247	17	9264	17	9281	17	9298	17	9315	17	9332	17	9349	16	9365	17	9382	17
580	3.9399	17	9416	17	9433	16	9449	17	9466	17	9483	16	9499	17	9516	17	9533	16	9549	17
590	3.9566	17	9583	16	9599	17	9616	16	9632	17	9649	16	9665	17	9682	16	9698	17	9715	16
600	3.9731	16	9747	17	9764	16	9780	17	9797	16	9813	16	9829	17	9846	16	9862	16	9878	16
610	3.9894	17	9911	16	9927	16	9943	16	9959	16	9975	16	9991	16	0007	17	0024	16	0040	16
620	4.0056	16	0072	16	0088	16	0104	16	0120	16	0136	16	0152	16	0168	16	0184	15	0199	16
630	4.0215	16	0231	16	0247	16	0263	16	0279	16	0295	15	0310	16	0326	16	0342	16	0358	15
640	4.0373	16	0389	16	0405	15	0420	16	0436	16	0452	15	0467	16	0483	15	0498	16	0514	16
650	4.0530	15	0545	16	0561	15	0576	16	0592	15	0607	16	0623	15	0638	15	0653	16	0669	15
660	4.0684	16	0700	15	0715	15	0730	16	0746	15	0761	15	0776	16	0792	15	0807	15	0822	15
670	4.0837	16	0853	15	0868	15	0883	15	0898	15	0913	15	0928	16	0944	15	0959	15	0974	15
680	4.0989	15	1004	15	1019	15	1034	15	1049	15	1064	15	1079	15	1094	15	1109	15	1124	15
690	4.1139	15	1154	15	1169	15	1184	14	1198	15	1213	15	1228	15	1243	15	1258	15	1273	14
700	4.1287	15	1302	15	1317	15	1332	14	1346	15	1361	15	1376	15	1391	14	1405	15	1420	15
710	4.1435	14	1449	15	1464	14	1478	15	1493	15	1508	14	1522	15	1537	14	1551	15	1566	14
720	4.1580	15	1595	14	1609	15	1624	14	1638	15	1653	14	1667	14	1681	15	1696	14	1710	15
730	4.1725	14	1739	14	1753	15	1768	14	1782	14	1796	15	1811	14	1825	14	1839	14	1853	15
740	4.1868	14	1882	14	1896	14	1910	15	1925	14	1939	14	1953	14	1967	14	1981	14	1995	14
750	4.2009	15	2024	14	2038	14	2052	14	2066	14	2080	14	2094	14	2108	14	2122	14	2136	14
760	4.2150	14	2164	14	2178	14	2192	14	2206	14	2220	14	2234	14	2248	14	2262	13	2275	14
770	4.2289	14	2303	14	2317	14	2331	14	2345	14	2358	14	2372	14	2386	14	2400	14	2414	13
780	4.2427	14	2441	14	2455	14	2469	13	2482	13	2496	14	2510	13	2523	14	2537	14	2551	13
790	4.2564	14	2578	14	2592	13	2605	14	2619	13	2632	14	2646	13	2659	14	2673	13	2686	14

**Table III:** 1958 *He*^4^ vapor pressure-temperature scale, *T* in ° K as a f unction of *P* in centimeters mercury at 0° C and standard gravity, 980.665 cm/sec^2^

*P*	0		1		2		3		4		5		6		7		8		9	
																				
80	4.2700	135	2835	133	2968	132	3100	131	3231	131	3362	129	3491	128	3619	127	3746	126	3872	125
90	4.3997	124	4121	123	4244	122	4366	122	4488	120	4608	120	4728	118	4846	118	4964	117	5081	116
100	4.5197	116	5313	114	5427	114	5541	113	5654	112	5766	112	5878	111	5989	110	6099	109	6208	109
110	4.6317	108	6425	107	6532	107	6639	106	6745	105	6850	105	6955	104	7059	103	7162	103	7265	102
120	4.7367	102	7469	101	7570	100	7670	100	7770	100	7870	98	7968	99	8067	97	8164	97	8261	97
130	4.8358	96	8454	96	8550	95	8645	94	8739	94	8833	94	8927	93	9020	92	9112	92	9204	92
140	4.9296	91	9387	91	9478	90	9568	90	9658	89	9747	89	9836	89	9925	88	0013	88	0101	87
150	5.0188	87	0275	86	0361	86	0447	86	0533	85	0618	85	0703	84	0787	84	0871	84	0955	83
160	5.1038	83	1121	82	1203	83	1286	81	1367	82	1449	81	1530	81	1611	80	1691	80	1771	80
170	5.1851	79	1930	79	2009	79	2088	78	2166	78										

**Table IV:** Temperature derivative, *dP/dT*, in millimeters *Hg*/° Kfor the 1958 *He*^4^ vapor pressure-temperature scale, *P* in millimeters mercury at 0° C and standard gravity, 980.665 cm/sec^2^

*T*	0.00	0.01	0.02	0.03	0.04	0.05	0.06	0.07	0.08	0.09

0.5	0.0005503	0.0007383	0.0009799	0.001287	0.001674	0.002157	0.002755	0.003489	0.004384	0.005468
.6	.006772	.008329	.01018	.01236	.01493	.01792	.02141	.02544	.03008	.03540
.7	.04148	.04838	.05622	.06506	.07501	.08617	.09863	.1125	.1280	.1451
.8	.1640	.1848	.2077	.2328	.2602	.2902	.3228	.3583	.3968	.4385
.9	.4835	.5320	.5843	.6406	.7008	.7654	.8346	.9084	.9871	1.071
1.0	1.160	1.255	1.355	1.462	1.575	1.694	1.820	1.953	2.093	2.240
1.1	2.395	2.557	2.728	2.907	3.095	3.291	3.496	3.711	3.935	4.169
1.2	4.412	4.666	4.930	5.205	5.491	5.788	6.096	6.416	6.747	7.090
1.3	7.445	7.813	8.194	8.587	8.993	9.413	9.846	10.29	10.75	11.23
1.4	11.72	12.22	12.73	13.26	13.81	14.37	14.95	15.54	16.15	16.77
1.5	17.41	18.06	18.73	19.42	20.12	20.84	21.58	22.33	23.10	23.88
1.6	24.68	25.50	26.33	27.18	28.05	28.94	29.84	30.76	31.69	32.64
1.7	33.61	34.59	35.59	36.61	37.64	38.69	39.76	40.84	41.94	43.05
1.8	44.18	45.33	46.49	47.67	48.86	50.06	51.28	52.52	53.77	55.03
1.9	56.30	57.59	58.89	60.20	61.52	62.85	64.19	65.54	66.91	68.28
2.0	69.65	71.03	72.42	73.81	75.20	76.60	77.99	79.39	80.79	82.20
2.1	83.60	85.00	86.38	87.74	89.09	90.41	91.71	92.96	94.28	95.77
2.2	97.31	98.88	100.5	102.1	103.7	105.4	107.1	108.8	110.5	112.2
2.3	113.9	115.7	117.5	119.2	121.0	122.9	124.7	126.5	128.4	130.3
2.4	132.1	134.1	136.0	137.9	139.9	141.8	143.8	145.8	147.8	149.8
2.5	151.9	153.9	156.0	158.1	160.2	162.3	164.5	166.6	168.8	171.0
2.6	173.2	175.4	177.6	179.8	182.0	184.3	186.6	188.8	191.2	193.5
2.7	195.9	198.2	200.6	202.9	205.3	207.7	210.1	212.5	215.0	217.4
2.8	219.9	222.4	224.9	227.4	230.0	232.4	235.0	237.6	240.1	242.7
2.9	245.4	248.0	250.6	253.3	256.0	258.6	261.3	264.1	266.8	269.5
3.0	272.3	275.1	277.9	280.7	283.5	286.3	289.2	292.0	294.9	297.8
3.1	300.7	303.7	306.6	309.5	312.5	315.5	318.5	321.5	324.5	327.6
3.2	330.6	333.7	336.8	339.9	343.0	346.1	349.3	352.4	355.6	358.7
3.3	361.9	365.1	368.4	371.6	374.8	378.1	381.4	384.7	388.0	391.3
3.4	394.6	398.0	401.3	404.7	408.1	411.5	414.9	418.3	421.7	425.2
3.5	428.7	432.1	435.6	439.1	442.7	446.2	449.7	453.3	456.9	460.5
3.6	464.1	467.7	471.3	474.9	478.6	482.3	486.0	489.7	493.4	497.2
3.7	500.9	504.6	508.4	512.2	516.0	519.8	523.6	527.5	531.3	535.2
3.8	539.1	543.0	546.9	550.8	554.8	558.8	562.8	566.8	570.8	574.9
3.9	578.9	583.0	587.1	591.2	595.4	599.5	603.7	607.9	612.1	616.3
4.0	620.6	624.8	629.1	633.3	637.6	642.0	646.3	650.6	655.0	659.4
4.1	663.7	668.1	672.6	677.0	681.4	685.9	690.3	694.8	699.2	703.7
4.2	708.1	712.6	717.1	721.6	726.1	730.7	735.3	740.0	744.7	749.4
4.3	754.1	758.9	763.7	768.6	773.4	778.3	783.2	788.1	793.0	797 9
4.4	802.9	807.9	812.9	817.9	822.9	828.0	833.1	838.1	843.2	848.4
4.5	853.5	858.7	853.9	859.1	874.3	879.5	884.8	890.1	895.3	900.7
4.6	906.0	911.4	916.7	922.1	927.6	933.0	938.5	943.9	949.4	955.0
4.7	960.5	966.1	971.7	977.3	982.9	988.6	994.4	1000	1006	1011
4.8	1017	1023	1029	1035	1041	1046	1052	1058	1064	1070
4.9	1076	1082	1088	1094	1100	1107	1113	1119	1125	1131
5.0	1137	1144	1150	1156	1163	1169	1175	1182	1188	1195
5.1	1201	1207	1214	1221	1227	1234	1240	1247	1254	1260
5.2	1267	1274	1280	………..	………..	………..	………..	………..	………..	………..

**Table V:** Auxiliary table for use in making hydrostatic head correction Table gives values of the ratio between the density of liquid He I at its saturated vapor pressure and the density of mercury at 0° C. (*P* in centimeters mercury at 0° C and standard gravity, 980.665 cm/sec^2^.) The density of mercury has been taken as 13.5951 g/cm^3^. If densities of liquid He I constitute critical data in an analysis, examination of the original literature is recommended.

*P*	0		1		2		3		4		5		6		7		8		9	
																				
0	0.01								0754	37	0717	35	0682	33	0649	30	0619	29	0590	29
10	.010561	27	0534	26	0508	26	0482	25	0457	24	0433	24	0409	23	0386	23	0363	23	0340	23
20	.010317	22	0295	22	0273	22	0251	21	0230	21	0209	21	0188	21	0167	21	0146	20	0126	21
30	.010105	20	0085	20	0065	20	0045	20	0025	20	0005	20	9985	20	9965	19	9946	20	9926	19
40	.009907	20	9887	20	9867	19	9848	20	9828	19	9809	20	9789	19	9770	20	9750	19	9731	20
50	.009711	19	9692	19	9673	20	9653	19	9634	20	9614	20	9594	20	9574	19	9555	19	9536	20
60	.009516	20	9496	19	9477	19	9458	20	9438	19	9419	20	9399	19	9380	20	9360	20	9340	19
70	.009321	20	9301	19	9282	19	9263	20	9243	19	9224	19	9205	19	9186	19	9167	20	9147	19
80	.009128	19	9109	19	9090	20	9070	19	9051	20	9031	19	9012	20	8992	20	8972	20	8952	20
90	.008932	19	8913	20	8893	20	8873	20	8853	20	8833	21	8812	20	8792	21	8771	20	8751	21
100	.008730	21	8709	22	8687	21	8666	22	8644	21	8623	21	8602	22	8580	22	8558	22	8536	23
110	.008513	23	8490	22	8468	23	8445	23	8422	24	8398	23	8375	24	8351	24	8327	25	8302	24
120	.008278	24	8254	24	8230	25	8205	26	8179	26	8153	26	8127	27	8100	26	8074	27	8047	28
130	.008019	28	7991	28	7963	28	7935	29	7906	29	7877	29	7848	30	7818	31	7787	32	7755	32
140	.007723	33	7690	32	7658	33	7625	34	7591	36	7555	36	7519	37	7482	38	7444	39	7405	41
150	.007364	42	7322	43	7279	44	7235	46	7189	48	7141	49	7092	51	7041	53	6988	56	6932	59
160	.006873	61	6812	64	6748	68	6680	73	6607	79	6528	87	6441	103	6338	130	6208	154	6054	190
170	.005864	229	5635	287	5348															

**Table VI:** Deviations of earlier scales from the 1958 scale, a*T_n_*-*T*_58_ in rnillidegrees

*n*	L55	55E	48	BS	37	32	29	24
*T_n_*								

° *K*								
0.7	−1.1	+ 1.0	+0.4	+0.3	………	−18.4	−2.7	−2.1
.8	−1.2	+ 1.1	+.5	+.3	………	−20.4	−10.3	−2.3
.9	−1.3	+1.2	+.6	+.4	−31.9	−22.1	−17.3	−2.4
1.0	−1.5	+1.3	+.3	+.4	−26.8	−23.5	−23.5	−2.6
1.1	−1.6	+ 1.4	−.2	+.4	−21.5	−24.7	−28.5	−2.7
1.2	−1.7	+ 1.5	+.4	+.5	−15.2	−25.4	−32.1	−2.8
1.3	−1.8	+ 1.5	+.3	+.6	−8.1	−25.7	−34.1	−3.0
1.4	−1.9	+ 1.6	+ 1.0	+.8	−2.3	−25.5	−34.5	−3.2
1.5	−2.0	+ 1.6	+ 1.3	+ 1.2	+1.1	−24.7	−33.2	−3.5
1.6	−2.1	+ 1.5	+2.9	+1.8	+2.6	−23.2	−30.3	+0.2
1.7	−2.1	+1.4	+3.8	+2.6	+3.2	−21.0	−25.9	+33.0
1.8	−2.2	+ 1.1	+3.9	+3.4	+3.5	−18.0	−20.2	+58.6
1.9	−2.2	+0.8	+5.1	+4.1	+4.2	−14.2	−13.3	+78.5
2.0	−2.2	+.6	+6.0	+4.6	+5.6	−9.7	−5.6	+93.6
2.1	−2.2	+.6	+8.4	+5.1	+7.1	−4.5	+2.5	+ 104.5
2.2	−2.2	+.8	+9.4	………	+9.3	+8.2	+8.2	+ 111.2
2.3	−2.1	+.5	+8.5	………	+8.2	+7.0	+7.0	+ 113.3
2.4	−2.0	0	+7.0	………	+6.9	+5.4	+5.4	+ 111.2
2.5	−1.9	−.4	+5.6	………	+5.5	+3.8	+3.8	+ 106.8
2.6	−1.8	−.7	+5.0	………	+4.4	+2.1	+2.1	+ 100.7
2.7	−1.5	−.9	+4.5	………	+3.6	+0.6	+0.6	+93.4
2.8	−1.3	−1.1	+3.6	………	+3.4	−.9	−.9	+85.2
2.9	−0.9	−1.1	+4.7	………	+3.6	−2.2	−2.2	+76.3
3.0	−.6	−1.1	+5.6	………	+4.2	−3.5	−3.5	+66.8
3.1	−.2	−1.1	+6.0	………	+5.3	−4.5	−4.5	+57.2
3.2	+.2	−0.9	+8.0	………	+6.7	−5.2	−5.2	+47.7
3.3	+.7	−.7	+8.6	………	+7.9	−5.7	−5.7	+38.4
3.4	+ 1.3	−.5	+8.7	………	+8.7	−5.9	−5.9	+29.4
3.5	+ 1.9	−.3	+8.8	………	+8.9	−5.8	−5.8	+21.0
3.6	+2.5	−.1	+8.7	………	+9.0	−5.5	−5.5	+13.2
3.7	+2.9	0	+8.6	………	+8.6	−4.8	−4.8	+6.2
3.8	+3.2	0	+7.7		+7.5	−3.9	−3.9	−0.1
3.9	+3.2	−.1	+6.6	………	+6.3	−2.6	−2.6	−5.4
4.0	+3.0	−.2	+5.0	………	+4.7	−0.9	−0.9	−9.6
4.1	+2.4	−.3	+2.6	………	+2.8	+1.2	+ 1.2	−12.7
4.2	+ 1.2	−.6	+0.5	………	+1.1	+3.7	+3.7	−14.6
4.3	0	−1.0	−.4	………	−0.5	+6.6	+6.6	−15.3
4.4	−.9	−1.4	+.9	………	………	+ 10.0	+10.0	−14.6
4.5	−1.5	−1.9	+3.8	………	………	+ 14.0	+14.0	−12.4
4.6	−1.7	−2.3	+7.7	………	………	+ 18.5	+18.5	−8.8
4.7	−1.5	−2.8	+ 11.9	………	………	+23.6	+23.6	−3.7
4.8	−0.9	−3.2	+ 15.8	………	………	+29.3	+29.3	+3.0
4.9	+.2	−3.7	+ 18.6	………	………	+35.7	+35.7	+ 11.1
5.0	+ 1.7	−4.1	+19.0	………	………	+42.6	+42.6	+20.8
5.1	+3.7	−4.5	+ 15.8	………	………	………	………	+32.1
5.2	+6.3	−5.0	+6.5	………	………	………	………	+45.0

a Explanatory notes concerning table of deviations of earlier scales from the 1958 scale:
*T*_24_: Defined by equation on p. 33 of Leiden Comm. No. 147b (Kamerlingh Onnes and Weber) and. by last equation on p. 23 of Leiden Comm. Suppl. No. 49 (Verschaffelt). These equations yield equal pressures at about 1.59° K. Therefore, values up through 1.5° K were derived from Ver schaffelt’s equation and those above from that of Kamerlingh Onnes and Weber. *T*_29_ Defined by eq (6) on p. 36 of Leiden Comm. No. 202c (Keesom, Weber, and Schmidt). These equations give equal pressures at 2.1765° K. The authors state in the last paragraph of the communication, p. 37, that the first of the equations fits reasonably well the data of Comm. No. 147b up to 5° K. Therefore, deviations up to 5.0° K have been included in the table. *T*_32_: Defined by the first of eq (6) cn p. 36 of Leiden Comm. No. 202c (Keesom, Weber, and Schmidt) and by the equation on p. 8 of Leiden Comm. No. 219a (Keesom). *T*_32_ and *T*_29_ are thus identical above the lambda point. These two equations are discontinuous by about 0.008° at 2.190° K and this fact was noted by Keesom (Leiden Comm. Suppl. 71d). *T*_37_: Defined by *T*_32_ together with curves in figure 1 of Leiden Comm. No. 250c (Schmidt and Keesom). Differences between *T*_37_ and *T*_32_ were determined diiectly from figure 1 with sufficient precision to determine differences between *T*_58_ and *T*_37_ to 0.1 millidegree. *T*_BS_: Defined by eq (9) and curve of figure 4, p. 1212 of Trans. Faraday Soc. **35** (Bleaney and Simon, 1939). Part, or all, of this scale is sometimes referred to as *T*_39_. In order to obtain differences between this scale and *T*_58_, pressures were calculated from the equation and curve with sufficient precision to yield differences to 0.1 millidegree. *T*_48_: Defined by tables I and II on pages T153 to T159 and by second equation on p. T152 of Procès-Verbaux des séances du Comité International des Poids et Mesures **23B**, T151 (1952). Values given in the table were obtained in the following ways. First, values of the difference between this scale and the 1958 scale were calculated at every 0.01° interval between 0.95° and 4.25° K from data of table II on pp. T158 and T159. The tabulated values from 1.0° through 4.2° K were then obtained by averaging the calculated differences between ***T*−** 0.05° and ***T+*** 0.05°, with weights of ½ assigned to the values at ***T−*** 0.05° and 74+0.05° and unit weights to all values at intermediate temperatures. For example, the tabulated value at 2.0° K is actually 1/20 of the sum of the differences at 1.95° and 2.05° plus 1/10 of the sum of the differences at 1.96°, 1.97°, 1.98°, 1.99°, 2.00°, 2.01°, 2.02°, 2.03°, and 2.04° K. The tabulated values at 0.7°, 0.8°, and 0.9° K were obtained by calculating the pressure corresponding to each 0.01° interval between 0.65° and 0.95° K from the data on p. T153 of table I and averaging the results in the manner just described. At 4.3° K and above, the tabulated values were obtained directly from the equation given on p. T152. Tables and equation have been published also by C. F. Squire, ***Low temperature physics***, pp. 229 to 233 and p. 26 (McGraw-Hill Book Co., Inc., New York, N.Y., 1953). *T*_55E_: Defined by the unnumbered equations on p. 188, ***Low temperature physics and chemistry*** (Clement), Proc. Fifth Intern. Conf. (Univ. of Wisconsin Press, Madison, Wis., 1958). Values of vapor pressure in millimeters mercury at 20° C were calculated by the computer at the U.S. Naval Research Laboratory (the NAREC) and values in millimeters mercury at 0° C were calculated by the computer at Los Alamos Scientific Laboratory (the MANIAC). The MANIAC calculation was used for obtaining the values in the table. *T*_L55_: Defined by table VII on p. 461, ***Progress in low temperature physics*** (Van Dijk and Durieux), (North-Holland Publishing Co., Amsterdam, Netherlands, 1957). Values below 0.9° K were obtained from the tables mentioned in section 23 of the reference. Table VII mentioned above is the same as table V published in Physica **24**, 1 (1958) and in Leiden Comm. Suppl. 113c.

**Table VII:** Auxiliary table for use in making corrections for density of mercury at temperatures other than 0°C^a^ Table gives values of the ratio between the density of mercury at the indicated temperature (° C) and that at 0° C.

*t°* C	0	1	2	3	4	5	6	7	8	9

10	0.99818	800	782	764	746	728	710	692	674	655
20	.99637	619	601	583	565	547	529	511	493	475
30	.99457	439	421	403	385	367	349	331	313	295

a Smithsonian Physical Tables, Ninth Revised Ed., edited by W. E. Forsythe, p. 153 (The Smithsonian Inst., Washington, D.C., 1954).

## Footnotes

1 Procès-Verbaux des séances du Comité International des Poids et Mesures 22, pp. 96 to 98, 114 to 118, 129 to 134 (1950).

2 G. D. Garland, pp. 221, 222, Karl Jung, p. 564, Handbuch der Physik edited by J. Bartels **47**, (Springer-Verlag, Berlin, 1956).

